# Demographic and Diagnostic Spectrum of Neurosurgical Biopsies: Initial Experience From a Re-established Neurosurgical Unit in a Tertiary Hospital in North Central Nigeria

**DOI:** 10.7759/cureus.35238

**Published:** 2023-02-20

**Authors:** Ijeoma Okwudire-Ejeh, Kevin N Ezike, Barnabas M Mandong, Ayuba M Dauda, Peter O Binitie, Danaan Shilong, Philip O Akpa

**Affiliations:** 1 Anatomic Pathology and Forensic Medicine, Asokoro District Hospital, Abuja, NGA; 2 Anatomic Pathology and Forensic Medicine, Nile University of Nigeria, Abuja, NGA; 3 Histopathology, Jos University Teaching Hospital, Jos, NGA; 4 Neurosurgery, Jos University Teaching Hospital, Jos, NGA; 5 Neurosurgery, Jos University of Nigeria, Jos, NGA

**Keywords:** nigeria, north central, cns, neurosurgical biopsies, neurosurgery

## Abstract

Introduction

Neurosurgical biopsies are obtained from lesions of the central nervous system, comprising the skull, brain, spine, spinal cord, and nerves. Neurosurgery practice is a highly specialized field with wide disparities related to access to care, especially in developing countries where there are few specialists and poor support care for patients. After over 20 years of redundancy, the neurosurgical unit in Jos University Teaching Hospital (JUTH), Jos, Plateau State, Nigeria, was re-established to meet the needs of patients in the area of neurosurgery.

The aim of the study is to document the demographic and diagnostic spectrum of neurosurgical biopsies obtained in JUTH in the first five years of the re-establishment of its neurosurgical unit, highlighting the need for inclusion of neurosurgical services in health planning and resource allocation; and to compare these findings to similar studies elsewhere.

Materials and methods

This was a retrospective, descriptive, hospital-based study of neurosurgical lesions diagnosed in the Department of Histopathology at JUTH between January 2011 and December 2015. One hundred and forty-five lesions met the inclusion criteria out of 151 in the records and were studied. Archival slides of these neurosurgical biopsies were retrieved, and fresh sections were re-cut and stained with hematoxylin and eosin (H&E) where necessary. The diagnoses of some of the neoplastic lesions were confirmed by immunohistochemistry. The data obtained was analyzed, and the results are presented as tables, bar charts, ratios, and percentages.

Results

Thirty-one different lesions were diagnosed. The lesions most commonly diagnosed were traumatic/degenerative intervertebral disc, 54/145 (37.2%); neoplastic, 48/145 (33.1%); and congenital, 31/145 (21.4%), while inflammatory/infectious, 9/145 (6.2%); and vascular, 3 (2.0%) lesions were the least. Bimodal peak frequencies involving the 0-14 years and 30-44 years age ranges were noted for the neoplastic lesions, occurring 37.5% (18/48) in the 0-14 years and 25% (12/48) in the 30-44 years, respectively. The 31 congenital anomalies diagnosed were all neural tube defects, and of these, occipital encephalocele, 10/31 (32.3%) and myelomeningocele, 9/31 (29.0%) were diagnosed most frequently. Of the neoplastic lesions, 66.7% (32/48) were benign and low-grade, and 33.3% (16) were malignant. Meningioma, 14/32 (43.8%), was the most common benign and low-grade neoplasm and accounted for 29.1% (14/48) of all neoplastic lesions. Astrocytoma (WHO grades I, II), 25% (8/32), was the next most common benign and low-grade neoplasm and accounted for 16.7% (8/48) overall. Astrocytoma (WHO grades III, IV), 8/16 (50%), was the most common malignant neoplasm and accounted for 16.7% (8/48) overall. Overall, neuroepithelial tumors, both benign and low-grade, and malignant, 43.8% (21/48), were the commonest neoplastic lesions. Most neoplastic lesions occurred in the brain, 75% (32/48), followed by the spine, 10.4% (5/48), and skull, 8.3% (4/48); while the least common was the spinal cord, 2.1% (1/48). The sex distribution of the neoplastic lesions showed almost equal frequency between males and females, 23/48 (47.9%) and 25/48 (52.1%).

Conclusion

The spectrum of neurological lesions highlighted in this study demonstrates that neurosurgical lesions abound in our environment with a similar prevalence to other regions of the world, and therefore speaks to the need for neurosurgical services.

## Introduction

Neurosurgical biopsies are taken from lesions involving the brain, spinal cord, nerves, skull, and spine; and include a wide spectrum of disorders such as tumors, brain hemorrhages, traumatic or non-traumatic blood clots, congenital diseases, and infections [1,2]. Neurosurgical lesions, irrespective of their diagnoses, can result in significant morbidity or mortality because of the structure of the central nervous system (CNS) and its functions [3,4]. Neurosurgery practice is a highly specialized practice requiring expertise and high-cost equipment, coupled with the need for prompt care and special training for caregivers; and it is almost non-existent in many areas, particularly resource-poor areas [5].

Neurosurgical lesions can be classified based on location into brain and spinal cord intra- and extra-axial lesions in relation to the involvement of the neuroaxis [6]. They can be further classified based on their behavior into benign and malignant lesions [7]. Regardless of whether they are benign, low-grade, or malignant, these lesions can result in adverse outcomes if prompt intervention is not instituted [8]. The global burden of neurosurgical lesions is unknown, and perhaps, because of the paucity of expertise, they constitute a small proportion of histological specimens in many areas, especially resource-poor areas [9]. Every year, an estimated 22.6 million patients suffer from neurological disorders or injuries that warrant the expertise of a neurosurgeon, of whom 13.8 million require surgery [10]. The global distribution varies from region to region also, with brain and spinal lesions occurring the most frequently and stroke, central nervous system (CNS) infections, and epilepsy occurring the least frequently [9,10]. The number of neurosurgeons and trained ancillary staff to the burden that neurosurgical lesions pose is wide, resulting in several deaths and disabilities in cases that should otherwise be treated [4,10]. The gap is even wider in developing countries of Asia and Africa, particularly among the rural populace [4]. The awareness of the diversity of neurosurgical lesions over the years has increased the need for more accurate and detailed differential diagnoses of the histology of intracranial and spinal space-occupying lesions (SOLs) [11].

After over 20 years of redundancy, the neurosurgical unit in Jos University Teaching Hospital (JUTH), Jos, Plateau State, Nigeria, was re-established in late 2010 to meet the needs of patients in the area of neurosurgery. The aim of this study, therefore, is to document the demographic and diagnostic spectrum of neurosurgical biopsies obtained in JUTH in the first five years of the re-establishment of its neurosurgical unit, highlighting the need for inclusion of neurosurgical services in health planning and resource allocation; and to compare these findings to similar studies elsewhere.

## Materials and methods

This study was a retrospective, descriptive hospital-based study of neurosurgical lesions diagnosed by histology at the Department of Histopathology of JUTH, Jos, Plateau State, North Central Nigeria, between January 2011 and December 2015. Case files of patients were retrieved from the medical records department, and patients’ biodata, clinical diagnoses, and clinical presentation were retrieved. The records of previous diagnoses, specimen histology numbers, age, sex, and site of the biopsy were retrieved from the histopathology registers and reconciled with the hospital case note records of the patients.

Archival slides of these neurosurgical biopsies were retrieved, and in the instances in which the slides were faded, fresh sections three to five micrometers thick were re-cut from paraffin wax-embedded, formalin-fixed blocks and stained with hematoxylin and eosin (H&E). Some of the neoplastic lesions diagnosed on histology were then subjected to immunohistochemistry to confirm their diagnoses. The 2021 WHO classification of CNS lesions in combination with specified diagnostic criteria was used to reclassify these neoplastic lesions.

Cases with missing tissue blocks or specimens with non-representative or inadequate tissue; or deficient clinical information with missing case folders were excluded from this analysis. Cytology or frozen section specimens were also not used. The data obtained were analyzed using Epi Info 7.2.0.1 (Centers for Disease Control and Prevention (CDC), Atlanta, Georgia, USA) and the Microsoft Office 365 version of Excel (Microsoft Corporation, Redmond, Washington, USA). The results are presented as tables, bar charts, ratios, and percentages.

Ethical clearance was obtained from the Institutional Research Ethics Committee of JUTH via a letter conveying this approval statement: Following a recommendation from the Institutional Research Ethics Committee, management has approved for you to proceed on your research topic, Morphological Pattern of Neurosurgical Biopsies at the Jos University Teaching Hospital: A Five-Year Retrospective Study.

## Results

**Figure 1:**
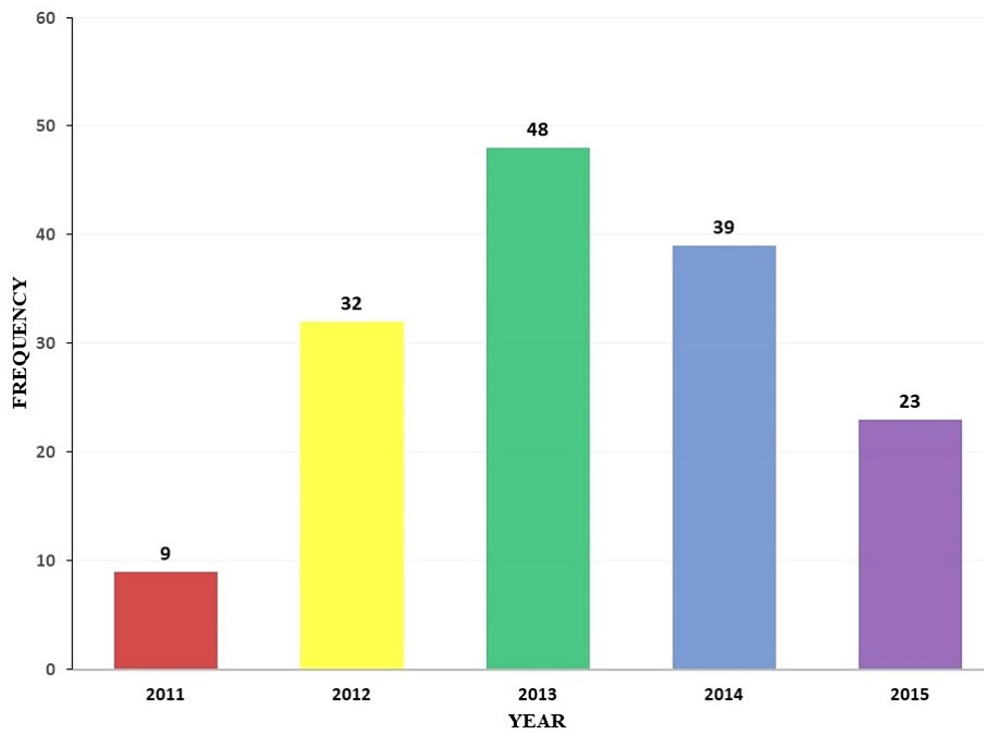
Yearly distribution of neurosurgical biopsies.

There were a total of 151 neurosurgical biopsies received at the histopathology laboratory of JUTH from January 2011 to December 2015 out of a total of 7948 biopsies, representing 1.9% of all the surgical pathology biopsy specimens received within the period of study. Over the five-year study period, there was a steady increase in biopsy numbers yearly for the first three years, 9, 32, and 48, respectively, and a decline thereafter to 39 and 23 in the next two years (Figure 1).

**Figure 2:**
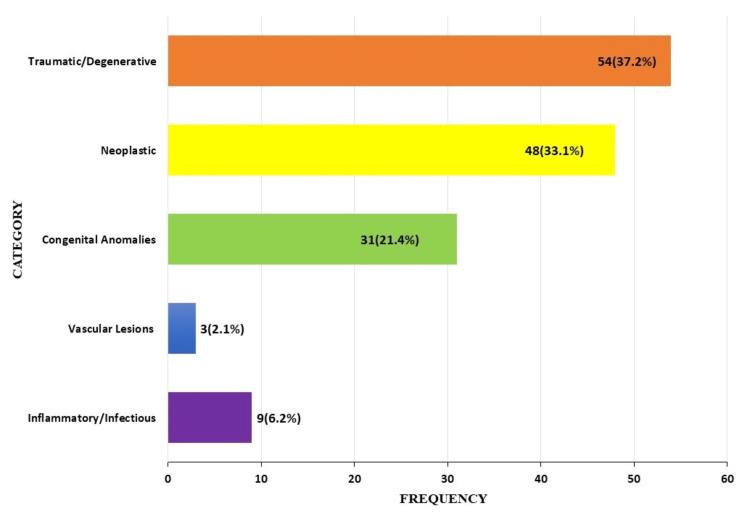
Diagnostic pattern of neurosurgical lesions.

Six out of the 151 biopsies did not meet the inclusion criteria, either due to tissue inadequacy or missing/incomplete clinical details, leaving 145 valid samples. A total of 31 different lesions were diagnosed, classified within five categories as follows: inflammatory/infectious, vascular, congenital anomalies, neoplastic, and traumatic/degenerative. Traumatic/degenerative lesions, 54/145 (37.2%); followed by neoplastic lesions, 48/145 (33.1%); and congenital anomalies lesions, 31/145 (21.4%) were the most frequently diagnosed; while inflammatory/infectious, 9/145 (6.2%); and vascular lesions, 3 (2.0%), were the least (Figure 2).

**Figure 3:**
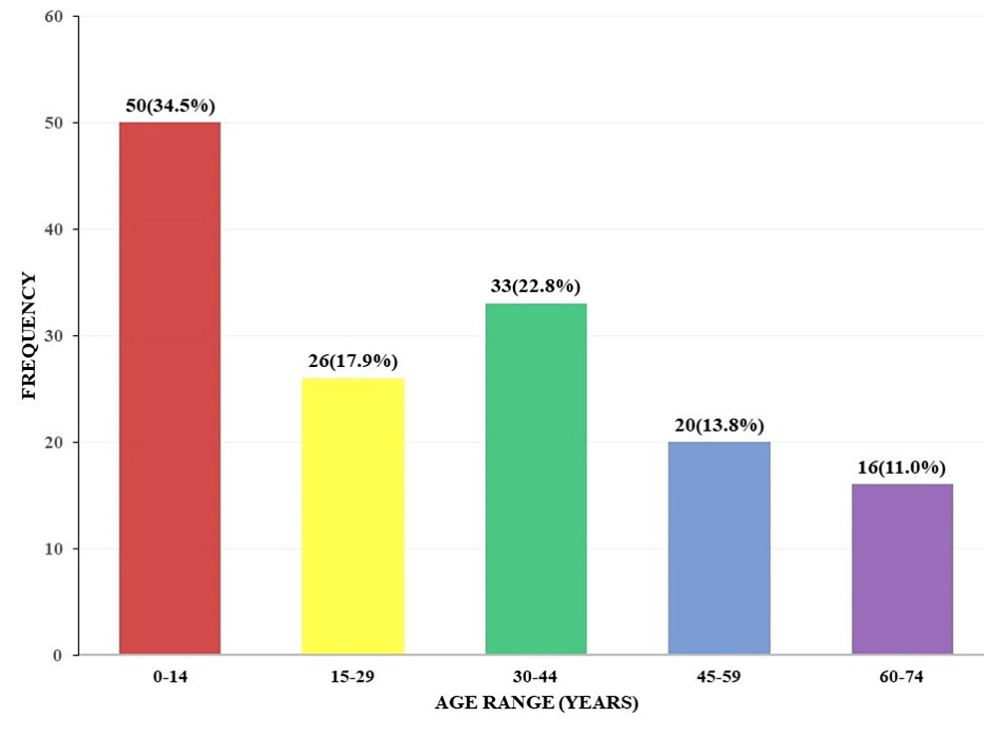
Age distribution of neurosurgical lesions.

The lesions are further grouped as non-neoplastic (inflammatory, vascular malformations, and traumatic/degenerative), congenital anomalies, and neoplastic. The age distribution of these lesions exhibits a somewhat bimodal peak frequency with 34.5% (50/145) seen in the 0-14 years and 22.7% (33/145) seen in the 30-44 years age ranges, respectively. In the 60-74 years age range, 11.1% (16/145) had the least number of cases (Figure 3).

Within the 0-14 years age range, however, a preponderance of lesions was seen in the 0-4 years age group, accounting for 28.9% (42/145) of overall cases. Within the second peak frequency of the 30-44 years age range, the 35-39 years age group had the highest frequency, accounting for 12.4% (18/145) of overall cases (Table 1). 

**Table 1:** Age distribution of neurosurgical lesions.

Age range (years)	Age group (years)	Frequency (n=145)		Percentage (%)	
0-14	0-4	42	50	28.9	34.5
	5-9	4		2.8	
	10-14	4		2.8	
15-29	15-19	8	26	5.5	17.9
	20-24	5		3.4	
	25-29	13		8.9	
30-44	30-34	10	33	6.9	22.7
	35-39	18		12.4	
	40-44	5		3.4	
45-59	45-49	10	20	6.9	13.8
	50-54	6		4.1	
	55-59	4		2.8	
60-74	60-64	11	16	7.6	11.1
	65-69	3		2.1	
	70-74	2		1.4	

Of the lesions categorized as non-neoplastic-inflammatory, vascular malformations, and traumatic/degenerative-the most frequent lesion diagnosed was traumatic/degenerative disc, accounting for 81.8% (54/66) while the least was cavernous hemangioma, 1.5% (1/66). The inflammatory lesions comprised a wide spectrum, with no particular lesion predominating. However, two cases each of brain abscess and tuberculosis were seen (Table 2).

**Table 2:** Diagnostic spectrum of non-neoplastic neurosurgical lesions.

Category	Diagnosis	Frequency (n=66)		Percentage (%)	
Inflammatory/infectious	Brain abscess	2	9	3.0	13.6
	Tuberculosis	2		3.0	
	Chronic osteomyelitis	1		1.5	
	Encephalitis	1		1.5	
	Epidural abscess	1		1.5	
	Mucocele	1		1.5	
	Subdural empyema	1		1.5	
Vascular lesions	Lobular capillary hemangioma	2	3	3.0	4.6
	Cavernous hemangioma	1		1.5	
Traumatic/degenerative	Degenerating intervertebral disc	54	54	81.8	81.8

**Figure 4:**
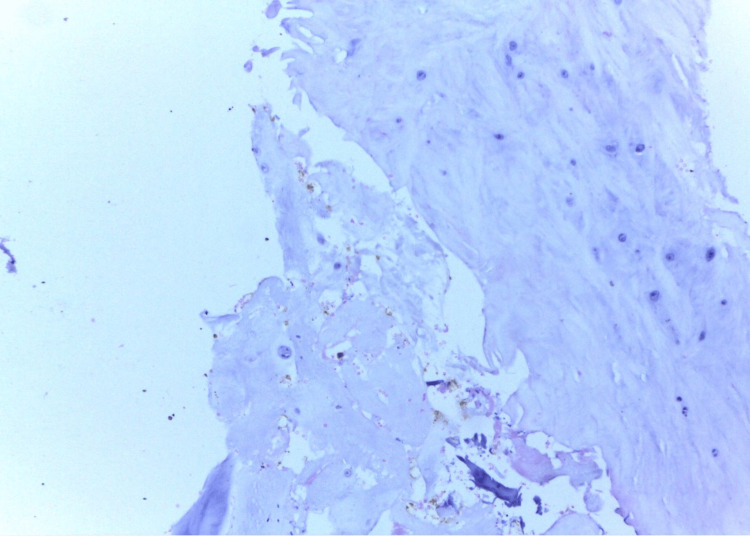
Degenerating/traumatic intervertebral disc from a 39-year-old male involved in a road traffic accident with trauma to C6-C7. Cartilage, fibrous tissue, and hemorrhage are seen. H&E ×400. H&E: hematoxylin and eosin.

Traumatic discs from the vertebral column were the majority of the traumatic/degenerative vertebral disc lesions (39/54), and this was due to trauma, including road traffic accidents and falls from heights, among others; while the rest were due to degenerative diseases (15/54). The photomicrograph shown and described below is a representative traumatic/degenerative vertebral disc lesion (Figure 4).

**Figure 5:**
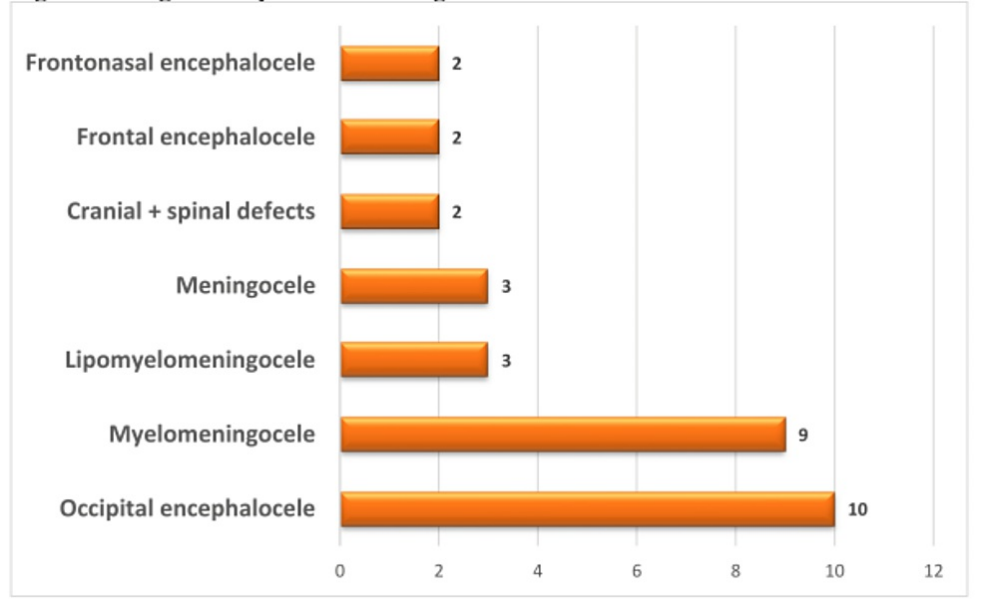
Diagnostic spectrum of congenital anomalies.

The 31 congenital anomalies diagnosed were all neural tube defects, and of these, occipital encephalocele, 10/31 (32.3%) and myelomeningocele, 9/31 (29.0%) were the most frequently diagnosed, while cranial + spinal defects, frontal encephalocele, and frontonasal encephalocele, 2/31 (6.5%) each were the least (Figure 5).

The 48 neoplastic lesions were further categorized as benign, low-grade, and malignant; and the benign and low-grade lesions, 32/48 (66.6%) were predominant. The most frequently diagnosed benign and low-grade neoplastic lesion was meningioma, accounting for 43.8% (14/32) and 29.1% (14/48) overall, respectively. Astrocytomas (WHO grades I, II) were the next most frequently diagnosed, accounting for 25% (8/32) of benign and low-grade, and 16.7% (8/48) overall. Astrocytomas (WHO grades III, IV) were the most common malignant neoplastic lesions, accounting for 50% (8/16), and 16.7% (8/48) overall (Table 3).

**Table 3:** Behavioral pattern of neoplastic lesions.

Category	Diagnosis	Frequency (n)		Percentage (%)	
Neoplasms (benign and low-grade)	Meningioma	14	32	29.2	66.7
	Astrocytoma (WHO grades I, II)	8		16.7	
	Lipoma	3		6.3	
	Mature teratoma	2		4.1	
	Neurofibroma	2		4.1	
	Hamartoma	1		2,1	
	Cementifying fibroma	1		2.1	
	Non-ossifying fibroma	1		2.1	
Neoplasms (malignant)	Astrocytoma (WHO grades III, IV)	8	16	16.7	33.3
	Ependymoma	2		4.1	
	Primitive neuroectodermal tumor (PNET)	2		4.1	
	Choriocarcinoma	1		2.1	
	Medulloblastoma	1		2.1	
	Plasmacytoma	1		2.1	
	Rhabdomyosarcoma	1		2.1	
	Total	48	48	100.0	100.0

Overall, in terms of tumor class, neuroepithelial tumors, both benign and low-grade, and malignant, including astrocytomas (WHO grades I-IV), ependymoma, PNET, and medulloblastoma, were the commonest neoplastic lesions, occurring in 43.7% (21/48) of cases. The meningeal tumors were next in frequency, occurring in 29.2% (14/48) of cases. Lipomas were the most common type of mesenchymal tumor, accounting for 14.7% (7/48) of all cases (Table 4).

**Table 4:** Histological classification of neoplastic lesions. PNET: primitive neuroectodermal tumor.

Tumor class	Histological subtypes	Frequency (n=48)		Percentage (%)	
Meningeal	Meningioma	14	14	29.2	29.2
Neuroepithelial	Astrocytoma (grades I, II)	8	21	16.7	43.7
	Astrocytoma (grades III, IV)	8		16.7	
	Ependymoma	2		4.1	
	PNET	2		4,1	
	Medulloblastoma	1		2.1	
Nerve sheath	Neurofibroma	2	2	4.1	4.1
Germ cell	Mature teratoma	2	2	4.1	4.1
Mesenchymal	Lipoma	3	7	6.3	14.7
	Cementifying fibroma	1		2.1	
	Hamartoma	1		2.1	
	Non-ossifying fibroma	1		2.1	
	Rhabdomyosarcoma	1		2.1	
Lymphoid	Plasmacytoma	1	1	2.1	2.1
Metastatic	Choriocarcinoma	1	1	2.1	2.1
	Total	48	48	100.0	100.0

**Figure 6:**
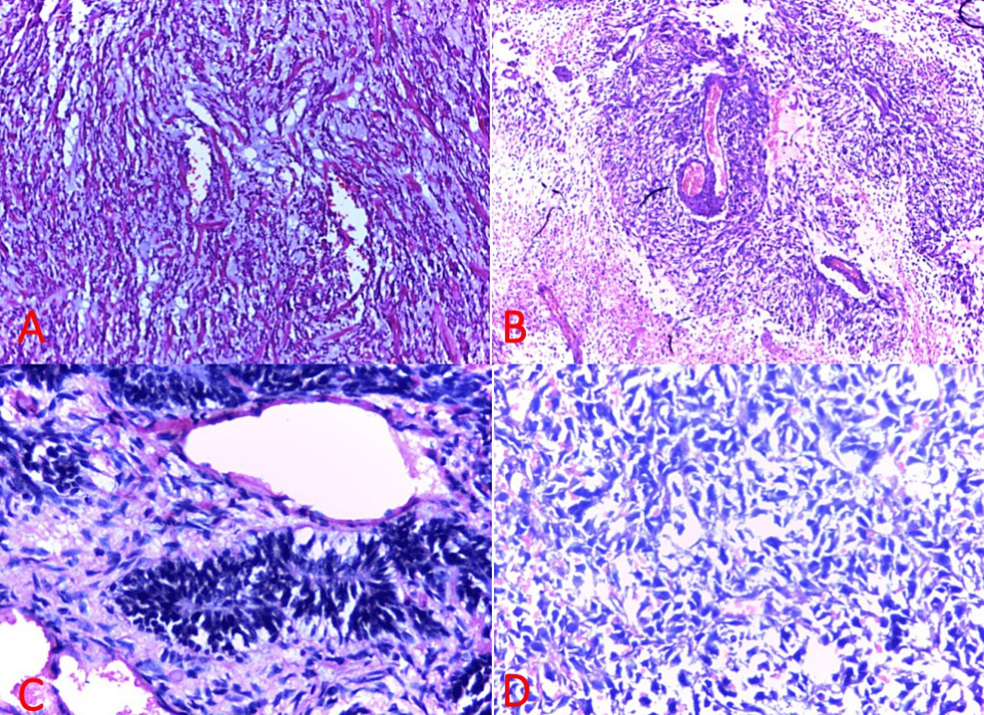
Neuroepithelial neoplastic neurosurgical lesions. (A) Pilocytic astrocytoma (WHO grade I) in a two-year-old girl with right facial nerve palsy. Microcystic spaces surrounded by astrocytes within a fibrillary background and a few Rosenthal fibers are seen. H&E ×200. (B) Glioblastoma (WHO grade IV) from a 17-year-old male presenting with headache, visual loss in the left eye, and paraparesis. Fibrillary background with extensive necrosis and atypical astrocytes arranged around blood vessels are seen. H&E ×400. (C) PNET from a four-year-old girl presenting with hemiparesis and visual loss. Atypical blue cells are seen with primitive neural tissue at the center. H&E ×400. (D) Medulloblastoma (WHO grade III) in a three-year-old girl presenting with ataxia. Tumor cells with scant cytoplasm are seen. Rosette formation is absent in this section. H&E ×400. H&E: hematoxylin and eosin, PNET: primitive neuroectodermal tumor.

In general, grade I astrocytomas predominate in young females, while higher-grade tumors (III-IV) predominate in teenage and older males. Of the other neuroepithelial tumors, two cases each of primitive neuroectodermal tumor (PNET) and medulloblastoma were seen; three of these occurred in female children below the age of four, while one (medulloblastoma) occurred in a male child. The only germ cell tumor seen, choriocarcinoma, was metastatic and occurred in a female. The photomicrograph shown and described below depicts four representative neuroepithelial neoplastic lesions (Figure 6).

**Figure 7:**
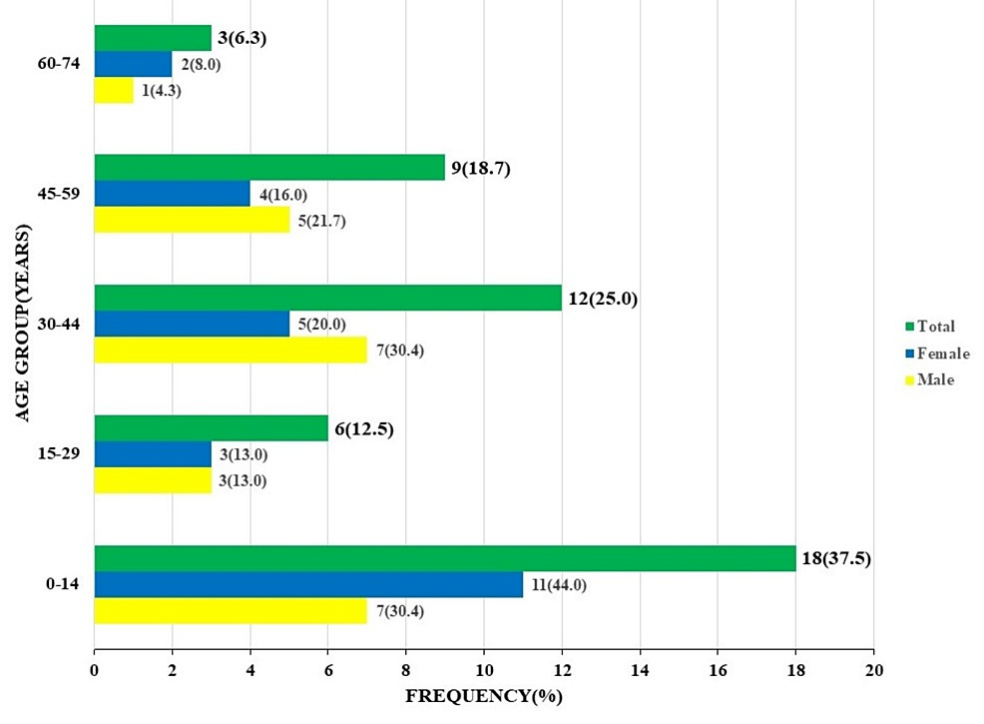
Age and sex distribution of neoplastic lesions.

The age and sex distribution of the neoplastic lesions showed almost equal frequency between males and females, 23/48 (47.9%) and 25/48 (52.1%). The highest frequency was seen in the 0-14 years age range, 18/48 (37.5%), with a male-to-female ratio of 1:1.5. A second peak was seen in the 30-44 years age range with 12/48 (25%) but showed a slight male predominance of 1.4:1 (Figure 7).

**Figure 8:**
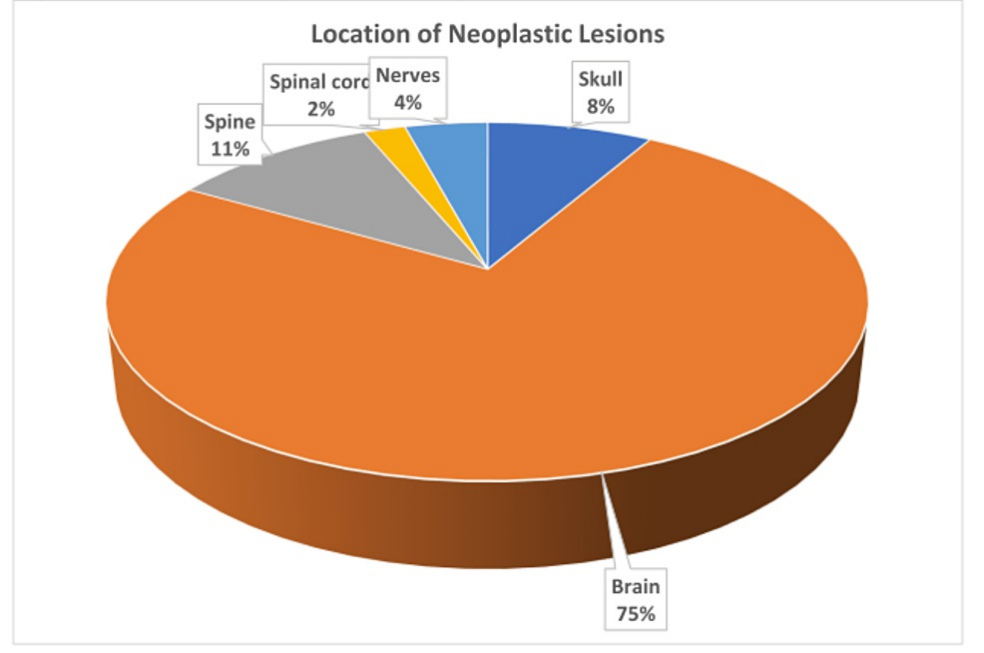
Location of neoplastic neurosurgical lesions.

The brain was the most frequent location of neoplastic lesions, accounting for 75% (32/48), while the spine, 10.4% (5/48) and the skull, 8.3% (4/48) followed. The least common location was the spinal cord (Figure 8).

## Discussion

In keeping with increasing awareness of its re-establishment, there was a steady yearly increase in the number of neurosurgical biopsies in the first three years, which declined in the fourth and fifth years due to service disruptions occasioned by industrial disputes rather than a reduction in the number of cases.

Traumatic/degenerative intervertebral disc was the most common diagnosis made in this study. It constituted 37.2% (54/145) of the entire diagnosis made. There is currently no data on the incidence of this lesion in Nigeria [12]. Different studies, mainly hospital-based, report varied rates of occurrence. For example, Ajiboye et al., in South-West Nigeria, reported an 11.5% annual incidence, and Edomwonyi et al., in South-South Nigeria, reported a prevalence of 3.5%. The differences may be due to disparities in institutional capacity for neurosurgical services [13,14]. Dewan et al. reported regional deficiencies in the number of practicing neurosurgical experts in Africa, resulting in a high proportion of untreated cases [10].

The age distribution of these lesions exhibits a somewhat bimodal peak frequency, with 50/145 (34.5%) seen in the 0-14 years and 33/145 (22.7%) seen in the 30-44 years age ranges, respectively. This differs from the findings of Schuhmann et al. [15], who reported a single peak frequency in the 30-39 years age group. The disparity could be due to the fact that our study had a preponderance of neoplastic lesions in the 0-14 years age range, while Schuhmann et al. had more traumatic brain injuries in the 30-39 years age range. 

The pattern of neurosurgical infections seen in this study is nonspecific and contrasts with the findings of Chapp-Jumbo et al. and Gora et al., who reported meningitis as the most frequent infection [16,17]. This could be due to the fact that the studies referenced above studied nonsurgical lesions while our study dwells mainly on neurosurgical biopsies. Spinal cord infections are very rare [18]. In keeping with this, there was no spinal cord infection recorded in this study.

Central nervous system congenital anomalies are under-reported in Nigeria and other developing countries [19]. However, congenital anomalies were a significant group of lesions in this study, comprising 21.4% (31/145). All 31 cases of congenital anomalies in this study were neural tube defects. This is in keeping with the worldwide trend but contrasts with the findings of Adeleye et al., who studied congenital CNS anomalies as a whole and reported a preponderance of congenital primary hydrocephalus over neural tube defects [20]. This may probably because in most cases of congenital hydrocephalus, neurosurgical interventions do not end up as biopsies. Of the 31 neural tube defects, occipital encephalocele, 10/31 (32.3%) and myelomeningocele, 9/31 (29.0%) were the most frequently diagnosed. Markovic et al. reported that occipital encephaloceles are the most common worldwide, a finding similar to this study [21]. However, other Nigerian studies have reported encephalocele as uncommon: Adetiloye et al. in South West Nigeria reported an incidence of 0.005/1000 live births in a multicenter study, while Shehu et al. in North West Nigeria reported no encephalocele in a hospital-based study that spanned 11 years [22,23].

Neoplastic lesions accounted for 33.1% (48/145) of the neurosurgical biopsies within the study period. This contrasts with Kumarguru et al., who reported a much higher percentage (84.4%) of neoplastic lesions in their cohort of neurosurgical cases [24]. A vast majority, 75% (36/48), of the neoplastic lesions in our study were intracranial, in keeping with findings by Zalata et al., who reported 86.7% frequency of intracranial as against 13.3% of spinal tumors [25]. Overall, intracranial neoplasms constituted 0.5% (36/7948) of all surgical pathology biopsies. This is a small proportion compared to the total number of surgical biopsy specimens, but higher than 0.004% of the total number of surgical biopsies reported by Soyemi et al [26]. Globally, spinal tumors are uncommon relative to the brain (intracranial) tumors and show minimal regional differences, ranging from 6.29/100,000 in high-income countries to 4.81/100,000 in middle- and low-income countries [27]. Our study, though hospital-based, recorded similar findings, with just 2.1% (1/48) of neurosurgical neoplasms occurring in the spinal cord. A similar study by Chikani et al. in South East Nigeria recorded 3.6% (17/472) of spinal procedures as primary spinal tumors [28].

There is a bimodal peak age of occurrence observed with the neoplastic lesions in our study as follows: 37% (18/48) in the 0-14 years and 25% (12/48) in the 30-44 years age ranges, respectively. These findings are similar to those of studies by Taha et al. in Saudi Arabia and Zhu et al. in China, but different from those of Thambi et al. and Lee et al., who reported single peak occurrences of 40-60 years and 60-70 years, respectively [29-32]. The male-to-female ratio of neoplastic lesions in our study was 1:1.1, very similar to the 1:1.1 and 0.9:1 reported by Soyemi et al. and Thambi et al., respectively [26,31]. Other studies report slightly higher ratios in favor of females [32,33].

The most common histological diagnosis of neoplastic lesions in our study was astrocytoma (WHO grades I-IV), which together accounted for 33.3% (16/48), while the next most common was meningioma, 29.2% (14/48). These findings are similar to those of Zalata et al., who reported gliomas as the most common, followed by meningiomas, and Soyemi et al., who also reported a slight predominance of astrocytoma over meningioma, but are at variance with several other studies, including those of Thambi et al. and Lee et al., who reported meningioma as the most common [25,26,31,32]. 

Intracranial cavernous malformations are uncommon, with a reported incidence of 0.15-0.56 per 100,000 persons [34]. The pattern observed in our study corresponds with the global trend.

In terms of overall incidence, metastatic tumors are the most common intracranial neoplasms, occurring up to 3-10 times more frequently than primary neoplasms [35]. In our study, however, the primary brain tumors predominated, with the only metastatic tumor seen being choriocarcinoma. This may be because most patients who develop brain metastases usually have multiple metastases in other sites and are often too ill for brain surgery, which in any case would be of doubtful benefit.

Limitations

The major limitations of the study were that it was hospital-based and had a small sample size. As a result, the findings may not reflect the true prevalence and incidence of neurosurgical lesions within the population. Other limitations were related to infrastructure and equipment lack and technical deficiencies. Being resource-challenged, we lack the wherewithal to carry out certain procedures such as polymerase chain reaction (PCR) for the detection of isocitrate dehydrogenase (IDH) mutations, which is required for proper classification of neoplastic neurosurgical lesions as per the current WHO guidelines.

## Conclusions

The spectrum of neurosurgical lesions highlighted in this study demonstrates that these lesions abound in our environment with a similar prevalence to other regions of the world, and therefore speaks to the need for neurosurgical services. With the expanding facility for neurosurgical services as well as the increase in the number of neurosurgeons at the Jos University Teaching Hospital, it is hoped that in the near future, more epidemiological studies would be undertaken in order to formulate policies for better and more affordable healthcare provision, particularly in neurosurgery.
